# The efficacy of Sustained Natural Apophyseal Glides with and without Isometric Exercise Training in Non-specific Neck Pain

**Published:** 2014

**Authors:** Abid Ali, Syed Shakil-ur-Rehman, Fozia Sibtain

**Affiliations:** 1 Dr. Abid Ali, Riphah College of Rehabilitation Sciences, Riphah International University, Islamabad, Pakistan.; 2 Dr. Syed Shakil-ur-Rehman, Riphah College of Rehabilitation Sciences, Riphah International University, Islamabad, Pakistan.; 3 Dr. Fozia Sibtain, Riphah College of Rehabilitation Sciences, Riphah International University, Islamabad, Pakistan.

**Keywords:** Sustained Natural Apophyseal Glides, Isometric Exercise Training, Non-specific Neck Pain

## Abstract

***Objective:*** To determine the efficacy of Sustained Natural Apophyseal Glides (SNAGs) with and without Isometric Exercise Training Program (IETP) in Non-specific Neck Pain (NSNP)

***Methods: ***This randomized control trial of one year duration was conducted at out-patient department of Physiotherapy and Rehabilitation, Khyber Teaching Hospital (KTH) Peshawar, Pakistan from July 2012 to June 2013. The sample of 102 patients of NSNP were randomly selected through simple random sampling technique, and placed into two groups. The SNAGs manual physical therapy technique with IETP was applied on 51 patients in group A and SNAGs manual physical therapy techniques was applied alone on 51 patients in group B. The duration of intervention was 6 weeks, at 4 times per week. The Neck Disability Index (NDI) and Visual Analog Scale (VAS) for neck pain were assessment tools used for all patients before and after 6 weeks of physical therapy intervention. All the patients were assessed through NDI and VAS before intervention and at the completion of 6 weeks program. The data of all 102 was analyzed by SPSS-20 and statistical test was applied at 95% level of significance determine the efficacy of both the treatments interventions and compare with each other.

***Results: ***The patients in group A, treated with SNAGs and followed by IETP for 6 weeks, demonstrated more improvement in pain and physical activity as assessed by VAS (p=0.013) and NDI (p=0.003), as compared to the patients treated with SNAGS alone, as pain and function assessed by VAS (p=0.047) and NDI (p=0.164). In group A the NDI score improved from 40 to 15 and VAS from 7 to 4, while in group B the NDI score improved from 42 to 30 and VAS from 7 to 4.

***Conclusion: ***Patients with non-specific neck pain treated with SNAGs manual physical therapy techniques and followed by IETP was more effective in reduction of pain and enhancement of function, as compared to those patients treated with SNAGs manual physical therapy techniques alone.

## INTRODUCTION

Neck pain or cervicalgia is a common neuro-musculo-skeletal problem, according to the available statistics with two-thirds of the population having neck pain at some point in their lives.^1^ The common symptoms of localized or radicular pain are tenderness, spasm, associated with functional disability. Neck pain is usually felt in the neck, but can be caused by numerous other spinal problems.^1^ Neck pain affects about 330 million people globally as of 2010 (4.9% of the population). It is more common in women (5.7%) than men (3.9%). It is less common than low back pain.^2^

Neck pain may arise due to bad postural, muscular tightness in both the neck and upper back, and pinching of the nerves emanating from the cervical vertebrae.^3^ Joints or muscles problems in the neck causes neck pain, upper back or upper extremity. It is usually diagnosed by physical examination, special orthopedic tests, X-rays, and MRI.^4^

Neck pain can be managed by conservative means including, medication interventional pain management techniques, and surgery. Physical therapy is an important component of conservative management of neck pain and post surgical rehabilitation after neck surgeries.^5^ The conservative physical therapy management includes; muscle strengthening, flexibility, and stabilization exercises, mobilization, manipulation, and mechanical traction procedures.^6^

There are different Mobilization techniques for neck pain, and mulligan’s technique is one of them. It has two techniques Sustain Natural Apophyseal Glides (SNAGS) and Natural Apophyseal Glides (NAGS).^7^ The aim of this study was to determine the efficacy of Sustained Natural Apophyseal Glides (SNAGs) with and without Isometric Exercise Training Program (IETP) in Non-specific Neck Pain (NSNP).

## METHODS

This randomized control study of one year duration was conducted at out-patient department of Physiotherapy and Rehabilitation, Khyber Teaching Hospital (KTH) Peshawar, Pakistan from July 2012 to June 2013. The sample of 102 patients of NSNP were randomly selected through simple random sampling technique, and placed into two groups. The study was approved by the institution review committee and a written informed consent was taken from all the study participants at the start of the treatment program.

The SNAGs manual physical therapy technique with IETP was applied on 51 patients in group A and SNAGs manual physical therapy techniques was applied alone on 51 patients in group B by a manual physical therapist skilled in both the techniques.

The duration of intervention was 6 weeks, at 4 times per week. The follow up of the patients were ensured through telephonic contact and no drop outs were observed during the study. All the patients were assessed through NDI and VAS before intervention and at the completion of 6 weeks program. The data of all 102 was analyzed by SPSS-20 and statistical test was applied at 95% level of significance determine the efficacy of both the treatments interventions and compare with each other.

## RESULTS

All 102 patients of non-specific neck pain, in both the groups had four weeks of physical therapy intervention. The patients in group A, treated with SNAGs and followed by IETP for 6 weeks, demonstrated better improvement in pain and physical activity as assessed by VAS (p=0.013) and NDI (p=0.003), in comparison to the patients in group B treated with SNAGS alone, VAS (p=0.047) and NDI (p=0.164). In group A the NDI score improved from 40 to 15 and VAS from 7 to 4, while in group B the NDI score improves from 42 to 30 and VAS from 7 to 4. (Table-I)

**Table-I:** Clinical and functional changes in all 102 patients at six weeks in patients with Non-specific neck pain (NSNP).

***Groups***	***Assessment Tools***	***Before Intervention*** ***(Group Mean Score)***	***After Intervention*** ***(Group Mean Score)***	***Significance*** ***(p-value)***
Group-A (n=51)	Neck Disability on NDI (total Score=50)	41	15	0.003
	Pain on VAS (Total Score=10)	07	02	0.013
Group-B (n=51)	Neck Disability on NDI (Total Score=50)	42	30	0.264
	Pain on VAS (Total Score=10)	08	04	0.147

## DISCUSSION

The results of this study demonstrated that the SNAGs and followed by IETP can better manage pain and disability as compared with the SNAGs alone in the management of Non-specific Neck Pain (NSNP). Brosseau and colleagues developed evidence-based clinical practice guidelines on therapeutic massage for neck pain on Ottawa panel and suggested that a multi-modal management strategy using mobilization or manipulation plus exercise is beneficial for relief of mechanical neck pain. Weaker evidence suggests less benefit to either manipulation/mobilization or exercise therapy done alone. This study strongly supports the result of our study.^9^

Bronfort and team conducted a randomized control trial to determine the relative efficacy of spinal manipulation therapy (SMT), medication, and home exercise with advice (HEA) for acute and sub-acute neck pain in both the short and long term. They concluded that for participants with acute and sub acute neck pain, SMT was more effective than medication in both the short and long term. However, a few instructional sessions of HEA resulted in similar outcomes at most time points.^10^

Tsakitzidis and colleagues carried out a systematic review to review (identify, critically appraise, and synthesize) the literature published on interventions for NS-NP and provide advice for health care providers to make evidence-based treatment decisions and to optimize their delivery of care for diagnosing, treating and managing adults with NS-NP. The history taking, excluding red flags and radicular pain/radiculopathy and assessing self-rated disability was deemed crucially important prior to selecting management and treatment modalities for NS-NP. They concluded that the evidence based practice is a multimodal approach including Manipulation/mobilization and supervised exercises.^11^

Gross and colleagues conducted a systematic review on Manipulation or mobilization for neck pain to determine the, and concluded that both the techniques had the same effects in neck pain.^12^ Miller and colleagues carried out a systematic review on Manual therapy and exercise for neck pain and concluded that Moderate quality evidence supports this treatment combination for pain reduction and improved quality of life over manual therapy alone for chronic neck pain.^13^

Leaver and colleagues conducted a randomized control trial on comparing manipulation with mobilization for recent onset neck pain and based on their results they concluded that the neck manipulation is not appreciably more effective than mobilization. The use of neck manipulation therefore cannot be justified on the basis of superior effectiveness.^14^

## CONCLUSION

Patients with non-specific neck pan treated with SNAGs manual physical therapy techniques and followed by IET is more effective in reduction of pain and enhancement of function, as compared to those patients treated with SNAGs manual physical therapy techniques alone.

## Authors Contribution:

**AA and SSR: **Conceived, designed and did statistical analysis & editing of manuscript.

**AA, SSR, and FS:** Did data collection and manuscript writing.

**SSR:** Did review and final approval of manuscript.
